# Liver abscess secondary to fishbone ingestion: case report and review of the literature

**DOI:** 10.1093/jscr/rjac026

**Published:** 2022-02-15

**Authors:** Niamh Grayson, Hiba Shanti, Ameet G Patel

**Affiliations:** Institute of Liver Studies, Kings College Hospital, London, UK

## Abstract

We report a rare silent migration of a fishbone into the liver and review the relevant literature. A 56-year-old man presented with a 2-day history of dull epigastric pain and raised inflammatory markers. Computerized tomography scan revealed a 4-cm abscess in the left lobe of the liver, with a linear radio-dense foreign body within the collection. At laparoscopy the hepatogastric fistula was disconnected. The fishbone was retrieved from the liver. Gastrostomy was closed with an omental patch. The patient had an uneventful recovery. Fifty-two cases of liver abscess secondary to enterohepatic fishbone migration were reported with over two-thirds presenting with a left-lobe abscess. There was marked variability in the management of liver abscess in the setting of fishbone migration-summarized in table. We believe that laparoscopic drainage of the abscess and extraction of the foreign body offer control of the source of sepsis and diminishes recurrence, whilst having a low-risk profile.

## INTRODUCTION

Foreign body ingestion is a common occurrence, majority of these pass without complications [1]. An estimated 1% of ingested foreign bodies result in gastrointestinal perforation, these are often sharp objects, such as accidentally ingested fishbones [2]. The sites of perforation vary, with the rectosigmoid or ileocolic being the most common [3].

We report a rare case of fishbone migration resulting in liver abscess and review of the literature. This was originally described in 1898 by Lambert [4].

## CASE PRESENTATION

**Figure 1:**
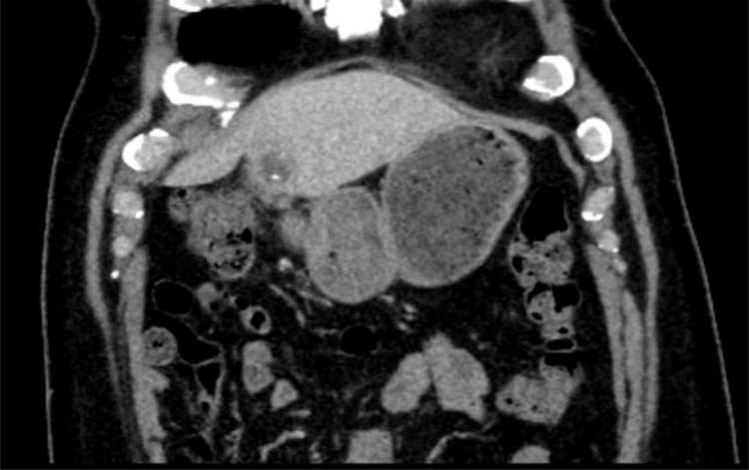
CT scan showing left lobe liver abscess with fishbone.

A 56-year-old man presented with a 2-day history of epigastric pain, leucocytosis and raised C Reactive Protein (CRP). A computed tomography (CT) scan revealed evidence of a 4.2 × 2.5 cm abscess in the left lobe of the liver (Segment III), with a linear radio-dense foreign body seen within the collection (Fig. 1). There was fat stranding around the pylorus. The patient was treated with antibiotics in his local hospital and a trial of aspiration revealed purulent fluid. An oesphagoduodenoscopy (OGD) was normal with no evidence of foreign body or inflammation in the stomach.

**Table 1:** Review of literature reported cases of enterohepatic fishbone migration

Author	Patient details	Symptoms	Duration (d)	Fishbone location	Fishbone size (mm)	Site of perforation	Management
Hernández-Villafranca, Spain, 2021 [26]	73 F	NA	14	Left lobe	30	Duodenum	Laparoscopic fishbone removal
Allam, UK, 2020 [9]	53 F	Pain, fever	7	Right lobe	NA	Pylorus	Antibiotics. Fishbone left *in situ*
Barkai, 2020, Israel [11]	66 F	Pain	NA	Right lobe	NA	NA	Laparoscopic fishbone removal
Burkholder, USA, 2019 [14]	64 F	NA	8	Left lobe	21	NA	Percutaneous abscess drainage. Fishbone left *in situ*
Bandeira-de-Mello, Brazil 2018 [10]	44 F	NA	14	Left lobe	25	Antrum	Laparoscopic fishbone removal
Bekki T, Japan, 2019 [13]	51 M	Fever	NA	Left lobe	24	Antrum	Laparoscopic fishbone removal
Beckers, Belgium, 2021 [12]	74 F	Pain, fever	3	Right lobe	35	NA	Laparoscopic fishbone removal
Goyal, USA, 2019 [25]	68 M	Pain, fever, WL	30	Left lobe	NA	Pylorus	Percutaneous abscess drainage. Fishbone left *in situ*
	56 M	Pain, fever	14	Right lobe	NA	Duodenum	Robotic fishbone removal
Li, China, 2019 [32]	58 M	Fever	9	Left lobe	40	NA	Laparoscopic left hepatectomy
Sim, Singapore, 2019 [42]	56 F	Fever, vomiting	2	Right lobe	NA	Stomach	Laparotomy and abscess drainage. Fishbone left *in situ*
Queiroz, Brazil, 2019 [40]	50 M	Pain, fever	10	Left lobe	NA	Antrum	Surgical removal
Yu, China, 2018 [49]	34 F	Pain, fever, vomiting	8	Left lobe	30	NA	Laparotomy and fishbone removal
Peixoto, Portugal, 2016 [38]	78 M	Fever, vomiting	2	Right lobe	35	NA	Laparotomy and fishbone removal
Venkatesan, Australia, 2019 [44]	88 F	Pain	60	Left lobe	NA	Antrum	Laparoscopic fishbone removal
Gómez Portilla, Spain, 2019 [24]	50 F	Pain, fever	28	Left lobe	25	NA	Left hepatectomy with bone removal
	69 F	NA	NA	Left lobe	NA	NA	Surgical removal
Chen, 2019, China [15]	37 M	Pain	60	Left lobe	17	NA	Liver resection
Mateus, Portugal, 2018 [34]	76 M	Pain	3	Left lobe	50	NA	Laparotomy and fishbone removal
	45 M	Weakness, chills	2	Right lobe	NA	NA	Percutaneous abscess drainage Fishbone left *in situ*
Fujiwara, Japan, 2017 [21]	69 M	Pain, fever	14	Right lobe	35	NA	Laparotomy with Fishbone removal
Dias, Brazil, 2018 [18]	35 M	Pain, fever	NA	Left lobe	25	NA	Laparotomy with fishbone removal
Lau, Singapore, 2017 [30]	85 F	Pain, fever	NA	Left lobe	40	Pylorus	Percutaneous fishbone removal
Tan, Singapore, 2016 [43]	56 M	Pain, fever	14	Left lobe	NA	Antrum	Laparoscopic Fishbone removal
	63 M	Fever	14	Left lobe	NA	Stomach	Laparoscopic Fishbone removal
Esseghaier, Tunisia, 2015 [20]	68 M	Pain, fever	7	Right lobe	20	Duodenum	Laparotomy with fishbone removal
Ede, South Africa, 2015 [19]	61 M	Pain, fever	21	Left lobe	60	NA	Laparotomy with fishbone removal
Panebianco, Italy, 2015 [37]	57 F	Pain, fever	14	Left lobe	40	Antrum	Laparoscopic Fishbone removal
Dinnoo, France, 2015	60 F	Pain, Sepsis	NA	Right lobe	NA	Duodenum	Laparoscopic fishbone removal
Xiao, China, 2015 [46]	47 F	Pain	365	Left lobe	25	NA	Laparoscopic fishbone removal
Venkatesh, Singapore, 2015 [45]	69 M	Pain, fever	5	Left lobe	14	Stomach	Left hepatectomy
Koşar, Turkey, 2014 [29]	73 F	Fever	NA	Left lobe	NA	NA	Laparoscopic fishbone removal
Dangoisse, Belgium, 2014 [17]	56 M	Fever, SOB	3	Left lobe	30	Stomach	Laparotomy with fishbone removal
Matrella, France, 2014 [35]	63 F	Pain, fever	10	Right lobe	40	NA	Laparotomy with fishbone removal
	83 F	Pain, fever	NA	Left lobe	NA	NA	Laparotomy with fishbone removal
Gaba, USA, 2013 [22]	33 F	Fever	14	Left lobe	30	NA	Percutaneous removal of fishbone
Masoodi, Saudi Arabia, 2012 [5]	45 M	Pain, fever	10	Right lobe	25	Duodenum	Laparotomy with fishbone removal
Jarry, France, 2011 [27]	68 F	Pain, fever	14	Right lobe	35	Duodenum	Laparotomy with fishbone removal
Liang, China, 2011 [33]	60 M	Pain, fever	30	Left lobe	27	Stomach	Surgical removal
Ng, Singapore, 2011 [36]	59 M	Fever	NA	Right lobe	NA	Pylorus	Antibiotics. Bone left *in situ*
Chen, China, 2011 [15]	59 F	Pain, fever	14	Left lobe	40	Duodenum	Liver resection
Yen, China, 2010 [48]	36 M	Pain, fever	14	Left lobe	NA	NA	Surgical removal
Santos, Portugal, 2007 [41]	62 F	Pain, fever	42	Left lobe	33	Antrum	Laparotomy and fishbone removal
Kadowaki, Japan, 2007 [28]	73 F	Pain, fever	7	Left lobe	28	NA	Laparotomy and fishbone removal
Clarençon, France, 2008 [16]	64 M	Pain, fever	NA	Right lobe	23	NA	Failed open surgical removal Percutaneous removal of fishbone
Perera, Sri Lanka, 2007 [39]	59 F	Pain	NA	Left lobe	45	NA	Laparotomy and fishbone removal
Lee, China, 2005 [31]	65 F	Pain, vomiting	7	Left lobe	35	Antrum	Laparotomy and fishbone removal
Goh, Singapore, 2005 [23]	32 M	Fever	5	Right lobe	30	Duodenum	Laparotomy and fishbone removal
Yang, China, 2005 [47]	40 M	Fever	7	Left lobe	50	NA	Percutaneous abscess drainage. Fishbone left *in situ*
Theodoropoulou, Greece, 2002 [7]	46 M	Pain, fever, jaundice	3	Left lobe	50	NA	Antibiotics. Fishbone left *in situ*
De la Vega, Spain, 2001 [6]	86 F	Pain, vomiting	NA	Right lobe	25	NA	Antibiotics. Fishbone left *in situ*
Horii, Japan, 1999 [2]	61 M	Fever	14	Left lobe	28	NA	Percutaneous abscess drainage and removal of fishbone.

The patient was transferred to our Hepatopancreaticobiliary (HPB) unit. On arrival, he was clinically well and asymptomatic. A repeat CT scan showed a persistent collection in the liver. On further enquiry, the patient revealed that a few weeks earlier as he had a transient episode of choking and discomfort whilst eating fish.

**Figure 2:**
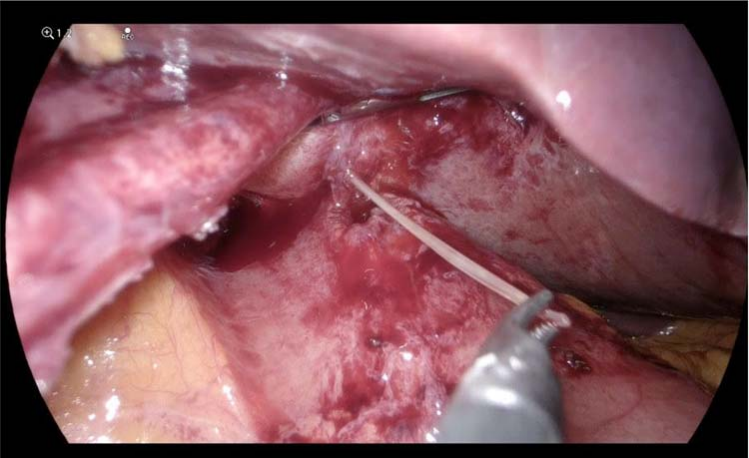
Fishbone extraction from the liver.

On laparoscopy, the left lateral segment of the liver was adherent to the gastric antrum (Fig. 2). Adhesions between the liver and stomach were divided with blunt and sharp dissection. The fishbone was pulled out of the liver intact and extracted through the port. The abscess was opened, drained and washed. A sealed fistulous tract was identified at the antrum; this was repaired with an omental patch. The patient had an uneventful recovery and was discharged the following day.

## DISCUSSION

Fifty-two cases of liver abscess secondary to enterohepatic fishbone migration have been reported in the English literature (Table 1). Most common symptoms included: anorexia, epigastric pain and fever. The lack of history of ingestion of a fishbone often leads to a diagnostic dilemma. CT scan was diagnostic in 47 that had axial imaging, three fishbones were found intra-operatively and two on autopsy. Over two-thirds of reported cases presented with a left lobe abscess, this is attributable to the anatomical proximity of the stomach.

There was marked variability in the management of liver abscess in the setting of fishbone migration. A variety of approaches including laparotomy, laparoscopy, CT guidance and liver resection were utilized to remove the fishbones. Percutaneous drainage usually results in the resolution of liver abscess, but recurrence is likely. Nine patients had the fishbone left *in situ*, one patient ultimately required a laparotomy for fishbone removal [5]. There were two mortalities in these patients with the fishbone left *in situ* (2/7, 29%), these were secondary to overwhelming sepsis, and the fishbones were discovered at autopsy [6, 7].

## LEARNING POINTS/TAKE-HOME MESSAGES

Left lobe liver abscess should raise the suspicion of a foreign body. Antibiotic treatment and drainage are effective in the short term. The retained foreign body acts as a nidus for recurrent infection and requires removal to prevent recurrence and mortality.

**Previous presentation:** Poster presentation in UGI conference 2021.

## CONFLICT OF INTEREST STATEMENT

None declared.

## FUNDING

Kings College London (JISC affiliated).

## References

[1] Abel RM , Fischer JE, Hendren WH. Penetration of the alimentary tract by a foreign body with migration to the liver. Arch Surg. 1971;102:227–8. 5544643 10.1001/archsurg.1971.01350030065021

[2] Horii K , Yamazaki O, Matsuyama M, Higaki I, Kawai S, Sakaue Y. Successful treatment of a hepatic abscess that formed secondary to fish bone penetration by percutaneous transhepatic removal of the foreign body: report of a case. Surg Today. 1999;29:922–6. 10489138 10.1007/BF02482788

[3] McCanse DE , Kurchin A, Hinshaw JR. Gastrointestinal foreign bodies. Am J Surg. 1981;142:335–7. 7283022 10.1016/0002-9610(81)90342-1

[4] Lambert A . Abscess of the liver of unusual origin. NY Med J. 1898;177–8.

[5] Masoodi I , Alsayari K, Al Mohaimeed K, Ahmad S, Almtawa A, Alomair A, et al. Fish bone migration: an unusual cause of liver abscess. BMJ Case Rep. 2012;2012:bcr0920114838. 10.1136/bcr.09.2011.4838 PMC3316812 22605588

[6] De la Vega M , Rivero JC, Ruiz L, Suarez S. A fish bone in the liver. Lancet. 2001;358:982. 11583753 10.1016/s0140-6736(01)06106-2

[7] Theodoropoulou A , Roussomoustakaki M, Michalodimitrakis MN, Kanaki C, Kouroumalis EA. Fatal hepatic abscess caused by a fish bone. Lancet. 2002;359:977. 10.1016/S0140-6736(02)07999-0 11918943

[8] Akhondi H , Sabih DE. Liver Abscess. Treasure Island (FL): StatPearls, 2021.

[9] Allam M , Pericleous S. Migrated fish bone induced liver abscess: medical management. Pan Afr Med J. 2020;36:140. 32849995 10.11604/pamj.2020.36.140.23783 PMC7422751

[10] Bandeira-de-Mello RG , Bondar G, Schneider E, Wiener-Stensmann IC, Gressler JB, Kruel CRP. Pyogenic liver abscess secondary to foreign body (fish bone) treated by laparoscopy: a case report. Ann Hepatol. 2018;17:169–73. 29311403 10.5604/01.3001.0010.7550

[11] Barkai O , Kluger Y, Ben-Ishay O. Laparoscopic retrieval of a fishbone migrating from the stomach causing a liver abscess: report of case and literature review. J Minim Access Surg. 2020;16:418–20. 31793447 10.4103/jmas.JMAS_196_19 PMC7597897

[12] Beckers G , Magema JP, Poncelet V, Nita T. Successful laparoscopic management of a hepatic abscess caused by a fish bone. Acta Chir Belg. 2021;121:135–8. 31433267 10.1080/00015458.2019.1658353

[13] Bekki T , Fujikuni N, Tanabe K, Amano H, Noriyuki T, Nakahara M. Liver abscess caused by fish bone perforation of stomach wall treated by laparoscopic surgery: a case report. Surg Case Rep. 2019;5:79. 31093821 10.1186/s40792-019-0639-0 PMC6520427

[14] Burkholder R , Samant H. Management of fish bone-induced liver abscess with foreign body left in situ. Case Reports Hepatol. 2019;2019:9075198. 31285930 10.1155/2019/9075198 PMC6594292

[15] Chen J , Wang C, Zhuo J, Wen X, Ling Q, Liu Z, et al. Laparoscopic management of enterohepatic migrated fish bone mimicking liver neoplasm: a case report and literature review. Medicine (Baltimore). 2019;98:e14705. 30882633 10.1097/MD.0000000000014705 PMC6426515

[16] Clarencon F , Scatton O, Bruguiere E, Silvera S, Afanou G, Soubrane O, et al. Recurrent liver abscess secondary to ingested fish bone migration: report of a case. Surg Today. 2008;38:572–5. 18516543 10.1007/s00595-007-3670-x

[17] Dangoisse C , Laterre PF. Tracking the foreign body, a rare cause of hepatic abscess. BMC Gastroenterol. 2014;14:167. 25262330 10.1186/1471-230X-14-167 PMC4190479

[18] Dias AR , Szor DJ, Ferreira CBA, Navarro CL. Uncommon cause of liver abscess. Clin Case Rep. 2018;6:1649–50. 30147930 10.1002/ccr3.1691 PMC6099009

[19] Ede C , Sobnach S, Kahn D, Bhyat A. Enterohepatic migration of fish bone resulting in liver abscess. Case Rep Surg. 2015;2015:238342. 26634168 10.1155/2015/238342 PMC4655044

[20] Esseghaier S , Nassej O, Haouas N, Benhassen I, Maamar AB, Daghfous MH. Liver abscess caused by migration of an ingested foreign body. Presse Med. 2015;44:851–3. 26051449 10.1016/j.lpm.2015.04.025

[21] Fujiwara Y , Shiba H, Nakabayashi Y, Otsuka M, Yanaga K. Hepatic abscess in the Spiegel lobe caused by foreign body penetration: report of a case report. Surg Case Rep. 2017;3:24. 28188512 10.1186/s40792-017-0297-z PMC5307392

[22] Gaba RC , Bui JT, Carroll RE. Catch of the day: forceps removal of embedded fish bone. J Vasc Interv Radiol. 2013;24:1545. 24070511 10.1016/j.jvir.2013.06.008

[23] Goh BK , Yong WS, Yeo AW. Pancreatic and hepatic abscess secondary to fish bone perforation of the duodenum. Dig Dis Sci. 2005;50:1103–6. 15986862 10.1007/s10620-005-2712-8

[24] Gomez Portilla A , Ezurmendia B, Martin E, Lopez de Heredia E, Muriel LJ. Fish bone-related intrahepatic abscess. An underdiagnosed condition? Cir Esp (Engl Ed). 2019;97:116–8. 30031548 10.1016/j.ciresp.2018.06.005

[25] Goyal P , Gupta S, Sapire J. Bone causing abdominal groans. J Emerg Med. 2019;57:e95–e7. 31378443 10.1016/j.jemermed.2019.06.013

[26] Hernandez-Villafranca S , Qian-Zhang S, Garcia-Olmo D, Villarejo-Campos P. Liver abscess due to a fish bone injury: a case report and review of the literature. Cir Cir. 2020;88:1–4. 10.24875/CIRU.20000030 33284283

[27] Jarry J , Nguyen V, Stoltz A, Imperato M, Michel P. A fish bone-related hepatic abscess. Clin Pract. 2011;1:e115. 24765356 10.4081/cp.2011.e115 PMC3981416

[28] Kadowaki Y , Tamura R, Okamoto T, Mori T, Mori T. Ruptured hepatic abscess caused by fish bone penetration of the duodenal wall: report of a case. Surg Today. 2007;37:1018–21. 17952539 10.1007/s00595-007-3524-6

[29] Kosar MN , Oruk I, Yazicioglu MB, Erol C, Cabuk B. Successful treatment of a hepatic abscess formed secondary to fish bone penetration by laparoscopic removal of the foreign body: report of a case. Ulus Travma Acil Cerrahi Derg. 2014;20:392–4. 25541855 10.5505/tjtes.2014.31643

[30] Lau CW , Wong KM, Gogna A. Image-guided percutaneous transhepatic removal of fish bone from liver abscess. J Radiol Case Rep. 2017;11:1–7. 10.3941/jrcr.v11i2.2997 PMC5443634 28580067

[31] Lee KF , Chu W, Wong SW, Lai PB. Hepatic abscess secondary to foreign body perforation of the stomach. Asian J Surg. 2005;28:297–300. 16234084 10.1016/S1015-9584(09)60365-1

[32] Li J , Zhao D, Lei L, Zhang L, Yu Y, Chen Q. Liver abscess caused by ingestion of fishbone: a case report. Medicine (Baltimore). 2019;98:e16835. 31441855 10.1097/MD.0000000000016835 PMC6716715

[33] Liang H , Liu OQ, Ai XB, Zhu DQ, Liu JL, Wang A, et al. Recurrent upper quadrant pain: a fish bone secondary to gastric perforation and liver abscess. Case Rep Gastroenterol. 2011;5:663–6. 22220141 10.1159/000335211 PMC3250653

[34] Mateus JE , Silva C, Beirao S, Pimentel J. Hepatic abscess induced by fish bone migration: two case reports. Acta Med Port. 2018;31:276–9. 29916359 10.20344/amp.9662

[35] Matrella F , Lhuaire M, Piardi T, Dokmak S, Bruno O, Maestraggi Q, et al. Liver hilar abscesses secondary to gastrointestinal perforation by ingested fish bones: surgical management of two cases. Hepatobiliary Surg Nutr. 2014;3:156–62. 25019078 10.3978/j.issn.2304-3881.2014.04.01 PMC4073319

[36] Ng CT , Htoo A, Tan SY. Fish bone-induced hepatic abscess: medical treatment. Singapore Med J. 2011;52:e56–8. 21451917

[37] Panebianco A , Lozito RC, Prestera A, Ialongo P, Volpi A, Carbotta G, et al. Unusual liver abscess secondary to ingested foreign body: laparoscopic management. G Chir. 2015;36:74–5. 26017106 PMC4469211

[38] Peixoto A , Goncalves R, Macedo G. Liver abscess associated sepsis caused by fish bone ingestion. GE Port J Gastroenterol. 2016;23:322–3. 28868489 10.1016/j.jpge.2016.03.006 PMC5580013

[39] Perera MT , Wijesuriya SR, Kumarage SK, Ariyaratne MH, Deen KI. Inflammatory pseudotumour of the liver caused by a migrated fish bone. Ceylon Med J. 2007;52:141–2. 18286779 10.4038/cmj.v52i4.919

[40] Queiroz RM , Filho FB. Liver abscess due to fish bone ingestion. Pan Afr Med J. 2019;32:26. 31143331 10.11604/pamj.2019.32.26.17822 PMC6522169

[41] Santos SA , Alberto SC, Cruz E, Pires E, Figueira T, Coimbra E, et al. Hepatic abscess induced by foreign body: case report and literature review. World J Gastroenterol. 2007;13:1466–70. 17457985 10.3748/wjg.v13.i9.1466 PMC4146938

[42] Sim GG , Sheth SK. Retained foreign body causing a liver abscess. Case Rep Emerg Med. 2019;2019:4259646. 31934467 10.1155/2019/4259646 PMC6942747

[43] Tan CH , Chang SY, Cheah YL. Laparoscopic removal of intrahepatic foreign body: a novel technique for Management of an unusual Cause of liver abscess--fish bone migration. J Laparoendosc Adv Surg Tech A. 2016;26:47–50. 26779724 10.1089/lap.2015.0487

[44] Venkatesan S , Falhammar H. Pyogenic hepatic abscess secondary to gastric perforation caused by an ingested fish bone. Med J Aust. 2019;211:451–e1. 31663145 10.5694/mja2.50395

[45] Venkatesh SH , Sanamandra SK. Large hepatic abscess caused by fish bone. Saudi Med J. 2015;36:878–9. 26108597 10.15537/smj.2015.7.11779 PMC4503912

[46] Xiao L , Li JW, Zheng SG. Laparoscopic extraction of a hepatic fish bone mimicking a liver mass after gastric perforation. Dig Dis Sci. 2015;60:2538–40. 25821100 10.1007/s10620-015-3637-5

[47] Yang CY , Kao JH, Liu KL, Chen SJ. Medical treatment of fish bone-related liver abscess. Clin Infect Dis. 2005;41:1689–90. 16267752 10.1086/498034

[48] Yen HH , Hsu YC. Education and imaging: gastrointestinal: pyogenic liver abscess associated with a penetrating fish bone. J Gastroenterol Hepatol. 2010;25:1900. 21155186 10.1111/j.1440-1746.2010.06551.x

[49] Yu W , Yu H, Ling J, Du J, Yin Z, Li C, et al. Hepatic abscess secondary to stomach perforation by a fish bone: a rare cause of hepatic abscess. Ann Hepatol. 2018;17:880–3. 30145566 10.5604/01.3001.0012.3171

